# Outpatient healthcare costs of childhood injuries in Australia: a 15-year longitudinal analysis using linked survey and health insurance data

**DOI:** 10.1186/s40621-025-00582-0

**Published:** 2025-11-18

**Authors:** Aquib M. Chowdhury, Kabir Ahmad, Rasheda Khanam

**Affiliations:** 1 Department of Critical Care, Barwon Health, Geelong, VIC Australia; 2 Department of Economics, Barwon Health, Geelong, VIC Australia; 3 Department of Economics, University of Southern Queensland, Toowoomba, QLD Australia

**Keywords:** Children, Healthcare cost, Medicare benefits scheme, Pharmaceutical benefits scheme, Childhood injuries

## Abstract

**Objective:**

This study evaluated the excess out-of-hospital healthcare costs associated with unintentional childhood injuries in Australia. This relationship was investigated within a longitudinal biennially surveyed cohort of 8,852 children aged 0–19 years. We assessed whether costs increased over age and with the duration of injury prevalence. Results were compared against cost estimates from similar studies in Australia and analogous developed nations.

**Data sources and study setting:**

The nationally representative Longitudinal Study of Australian Children provided linked Medicare Benefits Schedule (MBS) and Pharmaceutical Benefits Scheme (PBS) cost data for the Birth and Kindergarten cohorts, followed for 15 years.

**Study design:**

We used a mixed effects generalized linear model (GLM) with a gamma distribution and log link to estimate outpatient healthcare costs and assess the effect of injury status, controlling for sociodemographic characteristics. This model accounted for repeated measures over time and variability both within and between individuals.

**Results:**

Annual excess outpatient and pharmaceutical costs for injuries among 0–19 year-olds were A$39.1 million for those who were hospitalised and A$104.8 million for those only requiring community-based treatment. These estimates do not include inpatient hospital costs, which are not captured in the Medicare dataset.

**Conclusions:**

Unintentional childhood injuries in Australia incur significant financial burden on the public healthcare system, with costs per capita higher than other childhood conditions. Our figures are likely an underestimate. These excess healthcare costs support preventive efforts to reduce injury incidence among children.

**Supplementary Information:**

The online version contains supplementary material available at 10.1186/s40621-025-00582-0.

## Introduction

Unintentional injury is the foremost cause of morbidity and mortality in children and youths worldwide [1]. Of the 5.04 million deaths of children under 5 in 2020, the WHO estimated a sizeable proportion (336,000) was attributable to unintentional injury [2]. In children under the age of 18, over 850,000 deaths per annum are ascribable to unintentional injury worldwide, and it remains the cardinal cause of mortality in the 10–19 year age group [1].

Children are especially vulnerable to unintentional injury due to their incomplete cognitive and physical development [1, 3]. The unintentional injuries measured principally included incidents such as traffic injuries, submersion, poisoning, burns, and falls– although this list is non-exhaustive [1]. Younger children generally lack the ability to properly assess risk and avoid probable hazards [3], whereas older children become increasingly influenced by risk-taking behaviour [3].

Injury-related healthcare costs are substantial globally. Studies in the United States and New Zealand estimate billions in annual economic losses due to childhood injury, including direct medical and productivity costs [4–8]. Despite this, there is limited national-level data on the economic burden of childhood injuries in Australia using comprehensive longitudinal cohorts, even though national statistics show that child health outcomes remain a key policy concern [9].

Recent Australian studies offer important epidemiological and policy context for understanding this burden. For example, Aboriginal and Torres Strait Islander (ATSI) children have been shown to experience hospitalisation due to unintentional injuries at 1.7 times the rate of non-Indigenous children, highlighting inequities in injury risk and healthcare outcomes [10]. Falls are the most common cause of child injury hospitalisations in Australia, with one study estimating over 168,000 hospital admissions due to falls alone over a ten-year period, contributing to a total healthcare cost of AUD $2.1 billion [11]. Further, psychological impacts of injury are significant. One Australian study demonstrated that exposure to traumatic injury in early life can increase the risk of developing long-term psychological disorders, leading to elevated health service use and associated costs [12]. In addition, researchers have documented the prevalence and risk factors for low-speed vehicle run-over incidents—a preventable and often fatal mechanism of injury among young children in residential driveways [13].

This study aims to estimate the excess outpatient and pharmaceutical costs associated with unintentional childhood injuries in Australia using data from the Longitudinal Study of Australian Children (LSAC). We seek to further understanding of the development, wellbeing, and life trajectories of Australian children over time, providing a unique opportunity for long-term population-based analyses. We specifically used LSAC to evaluate injury incidence, costs, and trends across age groups, comparing hospitalised and non-hospitalised cohorts.

## Methods

### Study design and data source

This study draws on data from the Longitudinal Study of Australian Children (LSAC), a nationally representative, prospective cohort study designed to track the health, development, and wellbeing of children across Australia. LSAC is conducted by the Australian Institute of Family Studies in partnership with the Department of Social Services and the Australian Bureau of Statistics. The study follows two cohorts of children: the Birth (B) cohort, which began in 2004 with children aged 0–1 year, and the Kindergarten (K) cohort, comprising children aged 4–5 years at baseline.

Although most legal and policy frameworks in Australia define a child as an individual under 18 years of age, we included participants up to age 19 to maintain the full longitudinal follow-up of the LSAC cohorts. As the LSAC study design involves biennial data collection, children recruited at ages 0–1 or 4–5 were followed up into late adolescence, with the final wave (Wave 8) capturing some participants at age 19. Including age 19 ensures completeness of cost trajectories and captures injury-related healthcare use during a critical transition period from adolescence to adulthood. This approach is consistent with other longitudinal analyses using LSAC data.

Data are collected biennially through structured interviews with parents and caregivers, teacher questionnaires, and direct child assessments. Crucially, LSAC includes linked administrative health data from the Medicare Benefits Schedule (MBS) and Pharmaceutical Benefits Scheme (PBS), allowing for detailed analysis of publicly funded outpatient service use and prescription medications.

For this analysis, we used Waves 1 through 8 (2004–2018) for both cohorts, providing a longitudinal window spanning early childhood through late adolescence. The repeated-measures design allows for robust tracking of injury events, healthcare utilisation, and cost trajectories over time.

### Sample selection

The LSAC cohort was recruited using a two-stage clustered sampling design, in which children within the desired age brackets were randomly selected from the Medicare enrolment database, stratified by postcode to ensure national geographic representativeness. The sample was drawn from 311 postcodes across all Australian states and territories, covering urban, regional, and remote areas. Further detail on the LSAC sampling methodology is available in the technical documentation by Soloff et al. [14].

For this study, we included children with linked administrative health data available through Medicare—specifically, the Medical Benefits Schedule (MBS) and Pharmaceutical Benefits Scheme (PBS). These datasets capture government-subsidised outpatient and prescription medication use. Children with complete injury, cost, and relevant sociodemographic covariate data available across multiple waves were included in the analysis.

### Injury classification

Unintentional injuries were identified through parental reports during LSAC interviews. Parents were asked whether their child had experienced any accidents or injuries that required treatment from a doctor or hospital during the last 12 month since the interview time of each wave. Only for B cohort of Wave 1, the duration of injuries or accidents were considered since birth. The injury types or accidents reported by the parents were as follows: broken or fractured bones, burn or scald, dislocation, sprain or strain, cut or scrape, concussion or internal head injury, internal injury (not head), dental injury, accidental poisoning or other. Children were classified into three categories based on reported injury severity: (1) no injury, (2) injury without hospitalisation (treated in community settings), and (3) injury with hospitalisation (hospital presentation reported). This stratification enabled the evaluation of the healthcare cost gradient associated with differing levels of injury severity.

While LSAC does not consistently distinguish between unintentional and intentional injuries across all waves, specific questions on injury intent — such as whether the injury was accidental, intentional, or self-inflicted — were asked in later waves (e.g. B10, B12, B14 and K14, K16). Based on available responses and the broader developmental context, most reported injuries in childhood are likely to be unintentional. This interpretation aligns with prior Australian population-based studies using similar data sources.

### Outcome variable: healthcare cost

The primary outcome was total annual Medicare cost per child, including both MBS (outpatient services) and PBS (prescription medications) data. Costs were inflation-adjusted to 2018 Australian dollars. Cost estimates included population-level primary and secondary medical and pharmaceutical expenses, reflecting the structure of Australia’s mixed public-private healthcare system [15]. Importantly, inpatient hospital costs are not captured in MBS or PBS and are therefore excluded from our estimates.

### Statistical analysis

We used a mixed-effects generalized linear model (GLM) with a gamma distribution and log link to estimate excess healthcare costs associated with injuries. This method accounts for the right-skewed distribution of cost data and repeated measures within individuals over time [16–18]. Covariates included age, sex, presence of chronic conditions (obesity, asthma medication/wheezing presence, and disability/medical conditions), non-injury hospitalization status to adjust for healthcare utilization unrelated to injury, lagged injury counts from prior waves to account for potential delayed cost impact, parental education, family income, area remoteness, private insurance, and parental proactiveness on health seeking behaviour. The equation form of the mixed-effect GLM regression model and detail description of its covariates have been presented in Supplementary (Annex A).

Prior to model fitting, we conducted data exploration and pre-modelling checks, assessing continuous variables for distributional assumptions and outliers, and categorical variables for missingness and sparsity. Variance Inflation Factor (VIF) diagnostics indicated no multicollinearity concerns (mean VIF = 1.37; maximum VIF = 2.75). Marginal cost estimates and 95% confidence intervals were derived using post-estimation margins commands after the model-run. Post-estimation diagnostics, for example, Residual plots of deviance residuals vs. fitted values, assessment of the distribution of random effects, evaluation of model robustness through executing a 5-fold cross-validation procedure and computation of the root mean squared error (RMSE) across held-out folds were conducted to assess model assumptions.

### National cost projections

To estimate the national burden, marginal cost differences were multiplied by injury prevalence rates and applied to population data from the Australian Bureau of Statistics. This allowed us to generate age-stratified national-level estimates for both hospitalised and non-hospitalised injury cases.

### Sensitivity analysis

Sensitivity analyses were conducted using alternative model specifications, including a GEE model (gamma, log link), a fixed-effects panel regression with log-transformed costs, and a pooled GLM. All models consistently demonstrated a positive and statistically significant association between injury severity and increased healthcare costs, supporting the robustness of the primary mixed-effects GLM findings. Model comparisons were based on effect sizes, precision, and consistency across approaches. As only one observation had zero cost and was excluded, a two-part modelling for sensitivity analysis was not required.

## Results

### Prevalence of injuries by age

**Fig. 1:**
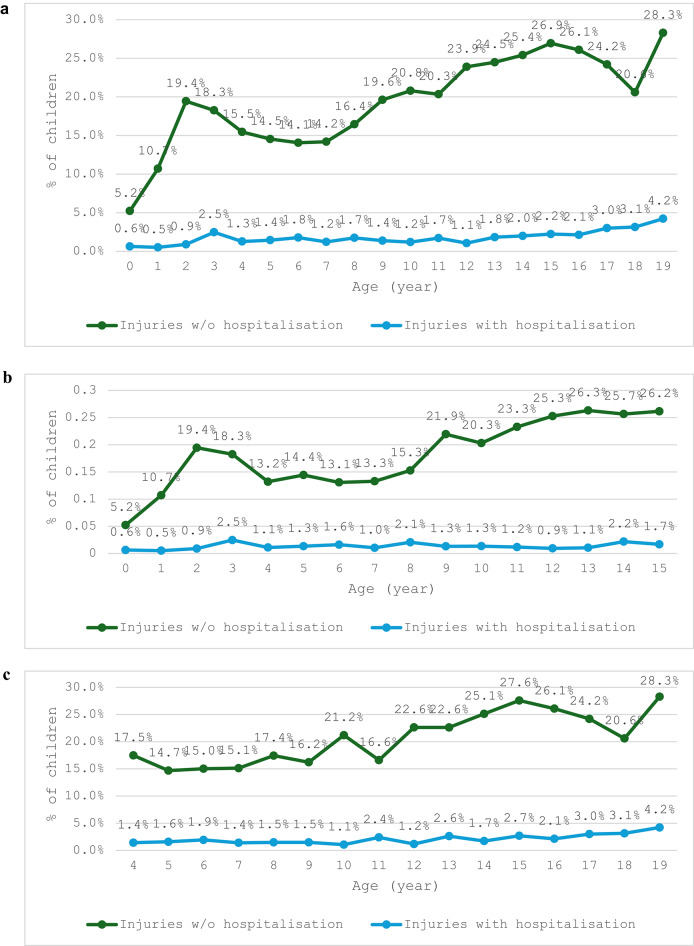
(**a**) Prevalence of injuries over age, combined cohort, 2004 to 2018. (**b**) Prevalence of injuries over age, B cohort, 2004 to 2018. (**c**) Prevalence of injuries over age, K cohort, 2004 to 2018. Source/Notes: Authors’ analysis of data from the Longitudinal Study of Australian Children

The prevalence of injuries without hospitalization varied significantly across different age groups, as revealed from the combined observations of both cohorts, ranging from 5.2% (95% CI: 4.7–5.7%) to 28.3% (95% CI: 26.8–29.8%) (Fig. 1a). This pattern followed a multimodal distribution, with prominent peaks at 3 and 15 years of age (19.4%, 95% CI: 18.1–20.7%; and 26.9%, 95% CI: 25.3–28.5%, respectively), and a smaller but notable peak at age 10 (22.5%, 95% CI: 21.1–23.9%). In contrast, the prevalence of injuries with hospitalization progressed linearly with age, ranging from 0.6% (95% CI: 0.4–0.8%) at age 0 to 4.2% (95% CI: 3.7–4.7%) at age 19. Cohort-wise prevalence rates were presented in Fig. 1b and c.

### Participant characteristics by injury type

Table 1 presents the characteristics of children by injury type, highlighting key differences in health and sociodemographic factors. Children who experienced injuries were more likely to have comorbid conditions such as asthma (18% in the hospitalised group vs. 11% in the non-injured group), disability, and overweight or obesity. Injured children also had higher rates of non-injury hospitalisations. Parental characteristics varied by injury type, with injured children more often having parents with lower educational attainment and reduced financial readiness to seek emergency healthcare—for example, 17% of parents in the hospitalised injury group reported low financial preparedness compared to 11% in the non-injured group. These patterns highlight the interplay between child health status, parental resources, and injury-related healthcare burden.

**Table 1:** Characteristics of the sampled children

Characteristics	All	By injury type of the children		
		No injury	Injuries without hospitalisation	Injuries with hospitalisation
Number of children in the first wave, B + K cohort (n)	8852	7798	967	87
Pooled number of observations (n)	51,448	41,092	9566	790
**Child characterisitics**				
Having any injury with or without hospitalisation (%)				
No Injury	79.2	-	-	-
Injury without hospitalisation	19.1	-	-	-
Injury with hospitalisation	1.7	-	-	-
Age, years (mean(SE))	9.1 (0.03)	8.7 (0.03)	10.4 (0.06)	10.7 (0.21)
Sex, male (%)	51.3	49.5	57.4	64.4
Any hospitalisation (at least one night) due to non-injury reason				
No	89.1	89.5	88.6	77.3
Yes	5.4	5.5	4.5	10.7
Missing (K cohort Wave 8)	5.5	5.1	6.8	12
Asthma medication use history				
No	84.2	84.8	82.3	78.8
Yes	15.8	15.2	17.7	21.2
Obesity category				
Underweight	6	6.2	5.4	5.2
Normal Weight	64.3	64.1	65.1	60.6
Overweight	17.5	17.2	18.3	22.5
Obese	8.9	9.1	7.9	8.3
Missing	3.3	3.3	3.3	3.4
Long-term disability or conditions by type				
No disability	85.4	86.1	83.1	75.3
Sensory	2	2.1	1.9	1.6
Physical	0.9	0.9	0.8	1.6
Psychological	0.6	0.5	1	0.8
Other long-term	3.1	3	3.2	4.1
Multiple (two or more of the above)	2.6	2.4	3.2	4.6
Missing (K cohort Wave 8)	5.5	5.1	6.8	12
**Parental charactisitcs**				
Single Parents (%)	18.1	17.6	19.9	23.3
Household Income Quintile				
* Quintile 1*	18.7	19.1	17.2	14.7
* Quintile 2*	18.6	18.6	18.3	17.8
* Quintile 3*	18.2	18.0	19.0	19.9
* Quintile 4*	18.4	18.1	19.4	18.7
* Quintile 5*	18.1	18.0	18.7	17.4
* Missing*	8.1	8.2	7.4	11.6
Employment status of parent 1				
* Employed*	68.7	67.6	72.9	72.4
* Unemployed*	3.0	2.9	3.2	3.0
* Not in labour force*	27.1	28.2	22.9	22
* Not disclosed*	1.2	1.2	1.0	2.6
Education of parent 1				
* Less than year 12*	17.6	17.8	16.5	20.4
* Year 12*	9.7	9.9	9	9.2
* Professional qualification/certificate course*	33.7	33.3	34.9	35.1
* Graduate/Diploma*	32.1	32.1	32.5	28.8
* Post-graduate*	6.9	6.9	7.1	6.5
Parents having any private hospital insurance				
* Yes*	45.4	45	47.2	42.6
* No*	54.6	55	52.8	57.4
Parental healthcare seeking proactiveness*				
High	59.4	60.2	56.5	52.4
Moderate	28.4	27.7	31	32.8
Low	10.7	10.5	11.5	12
No response	1.5	1.6	0.9	2.9
Residence Type				
* Major Cities*	67.9	68.5	66	62.1
* Inner region*	20.2	19.8	21.4	21.9
* Outer region*	10.4	10.1	11.1	14.4
* Remote/Others*	1.6	1.6	1.4	1.6
Total average healthcare costs ($) (mean (SE))				
MBS	389 (2.5)	367 (2.7)	454 (6.1)	725 (34.2)
PBS	46 (2.8)	43 (2.5)	55 (10.2)	81 (33.8)
Total Medicare**	434 (4.0)	409 (3.9)	509 (12.2)	806 (53.3)

MBS = Medicare Benefits Schedule; PBS = Pharmaceutical Benefits Scheme; SE = Standard Error. Total Medicare costs = MBS + PBS. Costs are in 2018 $AUD. All the mean and percentage figures are presented imposing population weight

### Statistical model outputs

Using a generalized linear model with a gamma distribution and log link (see supplementary Annex-B), we estimated that children who sustained injuries had significantly higher outpatient costs compared to those who did not (Table 2). Adjusted analyses controlled for confounding sociodemographic factors and allowed for repeated measures, thereby accounting for both between- and within-child variation over time to improve the accuracy of estimated injury-related cost differences. These findings remained robust across multiple sensitivity models, underscoring the substantial financial burden associated with childhood injuries in the Australian outpatient healthcare system.

**Table 2:** One-year total medicare costs ($ mean (SE)) per child by age-group and injury severity in the pooled observation of B and K cohort (*N* = 51448)

Age (years)	No of children by age group	GLM model results for mean total Medicare expenditure over two years					
		Mean (SE) by category of having injury and/or hospitalisation			Mean Diff. (95% CI) across the different injury categories by age of children		*P*-value
		No injury	Injuries without hospitalisaton	Injuries with hospitalisaton	Injuries WOH (B-A)	Injuries WH (C-A)	
		(A)	(B)	(C)			
0	3,943	392 (5)	481 (8)	741 (26)	89 (79–99)	349 (302–396)	< 0.001
1	718	409 (5)	502 (8)	774 (27)	93 (83–103)	365 (315–414)	< 0.001
2	2,802	371 (4)	455 (7)	701 (24)	84 (75–94)	330 (286–375)	< 0.001
3	1,106	378 (4)	464 (7)	715 (24)	86 (77–95)	337 (292–382)	< 0.001
4	6,338	347 (3)	426 (6)	656 (22)	79 (70–88)	309 (268–351)	< 0.001
5	1,679	356 (3)	437 (6)	673 (22)	81 (72–90)	317 (275–359)	< 0.001
6	4,740	349 (3)	427 (5)	658 (22)	79 (71–88)	310 (269–352)	< 0.001
7	2,059	350 (3)	429 (5)	662 (22)	80 (71–88)	312 (270–353)	< 0.001
8	4,410	348 (3)	427 (5)	658 (22)	79 (71–88)	310 (269–352)	< 0.001
9	2,102	361 (3)	443 (5)	683 (23)	82 (74–91)	322 (279–365)	< 0.001
10	3,844	354 (3)	434 (5)	668 (22)	80 (72–89)	315 (273–357)	< 0.001
11	2,255	369 (3)	452 (6)	697 (23)	84 (75–93)	328 (285–372)	< 0.001
12	3,280	363 (3)	445 (5)	686 (23)	83 (74–91)	323 (280–366)	< 0.001
13	2,551	374 (4)	459 (6)	707 (23)	85 (76–94)	333 (289–377)	< 0.001
14	3,307	385 (4)	472 (6)	727 (24)	88 (78–97)	343 (297–388)	< 0.001
15	1,899	386 (4)	473 (6)	729 (24)	88 (79–97)	344 (298–389)	< 0.001
16	1,270	355 (5)	435 (6)	670 (23)	81 (72–89)	316 (274–358)	< 0.001
17	997	369 (5)	452 (7)	696 (24)	84 (75–93)	328 (284–372)	< 0.001
18	1,312	512 (11)	628 (14)	968 (36)	116 (103–129)	456 (394–518)	< 0.001
19	836	530 (11)	650 (15)	1002 (37)	120 (107–134)	472 (408–537)	< 0.001
Total	51,448				1741	6819	

CI = Confidence Interval; SE = Standard Error. GLM model results presented using margins command after fitting a generalized linear model with gamma distribution and log link

#### Cost estimates by injury severity

The excess Medicare outpatient and pharmaceutical cost per child per year was A$116 without hospitalization and A$455 for children who had been hospitalised (Table 2). These values reflect increased medical utilization compared to their same aged peers who did not have any injuries, even in the absence of inpatient admission.

#### National-Level cost projections

Applying model estimates and prevalence data to population figures, we projected the annual cost burden for injury-related outpatient care. After projection, annual excess outpatient and pharmaceutical costs for injuries among 0–19 year-olds were A$39.1 million for those who were hospitalised and A$104.8 million for those only requiring community-based treatment (Table 3). These estimates do not include inpatient hospital costs, which are not captured in the Medicare dataset. These projections reveal that injuries in children result in significant fiscal implications for the healthcare system, particularly when aggregated across both hospitalised and non-hospitalised groups.

**Table 3:** Estimated total medicare costs ($) over 1 year for Australian population aged 0 to 19 years

Age (years)	Australian population	$ Costs in millions Estimate (95% CI) for children with:	Excess $ costs in millions above cost of non-injured children’s Estimate (95% CI) for children with:	
		No injuries	Injuries without hospitalisation	Injuries with hospitalisation
0	313,569	115.6 (113 to 118.2)	1.5 (1.4 to 1.5)	0.7 (0.6 to 0.8)
1	308,080	111.9 (109.4 to 114.3)	3.1 (2.9 to 3.2)	0.6 (0.5 to 0.6)
2	323,809	95.6 (93.6 to 97.5)	5.3 (5 to 5.6)	1.0 (0.8 to 1.1)
3	318,292	95.4 (93.5 to 97.2)	5.0 (4.7 to 5.2)	2.6 (2.3 to 3)
4	318,326	91.9 (90.4 to 93.4)	3.9 (3.6 to 4.1)	1.2 (1.1 to 1.4)
5	324,001	96.8 (95.3 to 98.3)	3.8 (3.6 to 4)	1.5 (1.3 to 1.7)
6	321,028	94.0 (92.6 to 95.4)	3.6 (3.4 to 3.8)	1.8 (1.6 to 2)
7	320,255	94.7 (93.4 to 96.1)	3.6 (3.4 to 3.8)	1.2 (1.1 to 1.4)
8	321,787	91.6 (90.3 to 92.9)	4.2 (3.9 to 4.4)	1.7 (1.5 to 1.9)
9	317,469	90.5 (89.2 to 91.9)	5.1 (4.8 to 5.4)	1.4 (1.2 to 1.6)
10	317,746	87.5 (86.2 to 88.8)	5.3 (5 to 5.5)	1.2 (1 to 1.3)
11	314,444	90.3 (88.8 to 91.7)	5.3 (5.1 to 5.6)	1.8 (1.6 to 2)
12	303,530	82.6 (81.3 to 83.9)	6.0 (5.7 to 6.2)	1.0 (0.9 to 1.2)
13	292,012	80.4 (79 to 81.8)	6.0 (5.8 to 6.3)	1.8 (1.6 to 2)
14	288,185	80.4 (78.9 to 81.9)	6.4 (6.1 to 6.7)	1.9 (1.7 to 2.2)
15	285,577	78 (76.5 to 79.5)	6.7 (6.4 to 7)	2.2 (1.9 to 2.4)
16	286,559	72.9 (71.2 to 74.5)	6.0 (5.7 to 6.3)	1.9 (1.7 to 2.1)
17	294,689	79 (77.1 to 80.8)	5.9 (5.7 to 6.2)	2.9 (2.6 to 3.2)
18	306,243	119.5 (114.7 to 124.3)	7.3 (6.9 to 7.7)	4.4 (3.9 to 4.9)
19	317,676	113.6 (109 to 118.2)	10.8 (10.2 to 11.4)	6.3 (5.6 to 7)
			104.8	39.1

CI = Confidence Interval; AUD = Australian Dollars. Estimates based on GLM marginal means and injury prevalence extrapolated to national population figures

### Additional results and supporting analyses

Further detailed results are provided in Table 4 and in the annexes to support and contextualise the main findings. Table 4 presents a comparison of government healthcare expenditure associated with childhood injury and other conditions based on previously published Australian studies. Annex C provides mean cost differences with 95% confidence intervals across injury categories by cohort and specific age groups. Annex D reports the marginal effects of injury-related healthcare costs across key covariate subgroups, illustrating variations in cost burden by sociodemographic and clinical factors. Lastly, Annex E presents the results of sensitivity analyses using alternative model specifications, confirming the robustness of our primary findings.

**Table 4:** Comparison of government healthcare expenditure in children associated with injury and other diseases adopted from different studies

Study	Medical condition	Age range and cohort	Study method	Excess costs per child (A$)		Excess costs at population level (A$m)	
				For the duration of the study (price year)	Per year in 2018 price	For the age range in the price year	For the age range in 2018 price
**Considering similar duration of other studies/diseases and this study**							
Sciberras et al. (2013)	ADHD	4/5 to 8/9 years–K cohort	GLM	2,245/6 years (2012)	421	12.4	13.9
Sciberras et al. (2013)	ADHD symptom	4/5 to 8/9 years–K cohort	GLM	753/6 years (2012)	138	15	16.9
Clifford et al. (2015)	Overweight	4/5 to 8/9 years–K cohort	OLS	164/6 years (2011)	31	6.9	7.8
Clifford et al. (2015)	Obese	4/5 to 8/9 years–K cohort	OLS	313/6 years (2011)	59	5.5	6.3
Au (2012)	Overweight/Obese	4/5 to 8/9 years–K cohort	GLM	93/5 years (2008)	23	9.8	12.1
Lucas et al. (2013)	Mental health	4/5 to 8/9 years–K cohort	GLM	909/4 years (2009)	277	4.7	5.7
Quach et al. (2013)	Sleep problem	4/5 to 6/7 years–K cohort	OLS	226/4 years (2012)	64	11	12.4
This Study	Injured without hospitalisation	4 to 9 years–B/K cohort	ME-GLM	480/6 years (2018)	80	24.2	24.2
This Study	Injured with hospitalisation	4 to 9 years–B/K cohort	ME-GLM	1880/6 years (2018)	313	8.8	8.8
**Considering whole duration of this study**							
	Injured without hospitalisation	0 to 19 years– B/K	ME-GLM	1741/15 years (2018)	116	104.8	104.8
This Study	Injured with hospitalisation	0 to 19 years– B/K	ME-GLM	6819/15 years (2018)	455	39.1	39.1

GLM = Generalized Linear Model; OLS = Ordinary Least Squares; MBS = Medicare Benefits Schedule; PBS = Pharmaceutical Benefits Scheme; AUD = Australian Dollars. All cost estimates adjusted to 2018 AUD values

## Discussion

### Interpretation of key findings

This study highlights the substantial outpatient and pharmaceutical costs associated with childhood unintentional injury in Australia, with a clear gradient by injury severity and age. Notably, costs were markedly higher for children who were hospitalised compared to those treated solely in the community. The identification of a multimodal distribution in injury prevalence—with prominent peaks at ages 3, 10, and 15—underscores age-specific vulnerabilities.

### Developmental explanations

The observed age-related trends can be explained through developmental and behavioural lenses. Early childhood is marked by limited coordination and poor hazard perception, while adolescence features increased independence and risk-taking behaviour due to ongoing neurological development, particularly in the prefrontal cortex [3]. The smaller but notable peak at age 10 may reflect increased unsupervised physical activity, organised sports participation, and limited risk awareness characteristic of late childhood.

### Comparison with international literature

Our cost estimates were modest compared to those reported in other high-income countries. For instance, US-based studies report injury-related healthcare costs that are three to four times higher after adjusting to Australian dollars [6, 8]. These differences may reflect variations in healthcare systems, injury definitions, and methodological approaches. Nonetheless, the international comparisons validate the economic relevance of our findings.

### Comparison with other childhood conditions

When benchmarked against other common childhood health conditions in Australia, the excess healthcare costs associated with unintentional injury were comparatively high. For instance, the annual Medicare cost of ADHD symptoms was estimated at approximately A$138 per child, and overweight or obesity were associated with additional annual costs of A$31 and A$59 per child, respectively [19–21]. More recent IV-based estimates suggest this burden may be even higher [22]. Similarly, childhood sleep disorders have been associated with additional primary care costs of approximately A$64 per child annually by age 7, further contextualising the outpatient burden of preventable conditions [23]. Mental health difficulties during childhood have been estimated to incur excess annual Medicare costs of approximately A$277 per child [24].

In contrast, our findings show excess Medicare costs of A$105 for children with injuries treated in the community and A$487 for those hospitalised. These comparisons highlight that injury-related healthcare costs may exceed those of other chronic conditions during childhood. Given that many unintentional injuries are preventable, this reinforces the importance of targeting injury prevention as a priority area in health policy and child development strategies.

### Policy implications

The national-level cost projections (Table 3) reinforce the significant fiscal burden of childhood injury on Australia’s healthcare system. These findings suggest a clear need for enhanced injury prevention strategies tailored to high-risk age groups, and especially in the domestic environment where injuries are more likely to occur [25]. Public health initiatives, school-based safety programs, and parental education could yield long-term cost savings and improve health outcomes.

### Strengths and limitations

Key strengths of this study include the use of a nationally representative longitudinal cohort (LSAC), and linkage to administrative MBS and PBS data, allowing for robust cost estimation of outpatient services use and prescription medications. A key limitation of this study is the exclusion of inpatient hospital costs from the linked Medicare data, which captures only outpatient services and prescription medication use. Consequently, total injury-related healthcare expenditures are likely underestimated, particularly for injuries requiring hospitalisation, and absence of indirect costs such as parental work loss or long-term disability. Although external sources such as the AIHW Injury in Australia report [26], the NHCDC [27], and AR-DRG cost weights [28] provide estimates of hospital costs and injury case numbers separately, they are not directly linked. Integrating these sources would require strong assumptions about injury types, hospital service use, and case severity, which cannot be validated with the LSAC data. Nevertheless, this study offers important insights into the outpatient cost burden of childhood injury, and future research incorporating hospital separation and diagnosis data could enhance total cost estimations.

The LSAC sampling frame excluded children living in very remote areas and some discrete Indigenous communities. As a result, Aboriginal and Torres Strait Islander (ATSI) children living in these regions may be underrepresented in the sample. However, ATSI children residing in urban and regional areas were eligible and are included in the dataset. This limitation should be considered when generalising findings to all Indigenous populations in Australia.

## Conclusion

The current study provides evidence of a significant relationship between injury among Australian children aged 0–19 years and major excess healthcare costs at the population level, estimated approximately A$94.7 million for non-hospitalised injuries and A$41.0 million for hospitalised injuries, annually. Public health spending could be significantly reduced by a successful campaign to prevent injuries in early childhood years of life. These research results will also assist health economists to estimate the cost of childhood injury from the healthcare services and medication perspective when conducting modelled economic evaluations of childhood injury.

## Electronic supplementary material

Supplementary Material 1

## Data Availability

No datasets were generated or analysed during the current study.

## Declarations

### Precis

Regression analysis of the public outpatient healthcare costs from a longitudinal sample.
