# Supplementary material for: Outpatient healthcare costs of childhood injuries in Australia: a 15-year longitudinal analysis using linked survey and health insurance data
Source: Inj Epidemiol. 2025 Nov 18;12:77. doi: 10.1186/s40621-025-00582-0 (PMC12625061; doi:10.1186/s40621-025-00582-0)
Supplement: Supplementary file 1 — Supplementary Material 1 [file 40621_2025_582_MOESM1_ESM.docx]

# Annex A: Study Model Specifications

## Annex A1: Mixed-Effects GLM Model Specification

The following represents the regression equation used in the mixed-effects generalised linear model (GLM) with a gamma distribution and log link, where the outcome variable is total annual Medicare cost per child:

log(E[Y_it]) = β₀ + β₁Injury_it_ + β₂Gender_i_ + β_3_Age_i_ + β_4_Cohort_i_ + β_5_LaggedInjury_it_ + β_6_Non-InjuryHospitalisation_it_ + β_7_Asthma_it_ + β_8_Obesity_it_ + β_9_DisabilityMedcon_it_ + β_10_ParentalProactiveness_it_ + β_11_PrivateInsurance_i_ + β_12_Remoteness_it_ + β_13_SingleParent_it_ + β_14_SES_it_ + u_i_

Where:

Y_it_: Annual Medicare cost for child i at time t

Injury_it_: Indicator of injury severity (none, non-hospitalised, hospitalised)

Gender_i_: Gender of the child

Age_i_: Age of the child

Cohort_i_: Cohort membership (B or K)

LaggedInjury_it_: Categorical variable for prior injuries

Non-InjuryHospitalisation_it_: Categorical variable for whether hospitalised for non-injury reasons

Asthma_it_, Obesity_it_, DisabilityMedCon_it_: Indicators for comorbid conditions (asthma, obesity, disability/long-term medical condition)

ParentalProactiveness_it_: Proxy for healthcare-seeking behaviour related to medical emergency services

PrivateInsurance_i_: Private hospital insurance status

Remoteness_it_: Geographic classification (e.g., urban, regional, remote)

SingleParent_it_: Whether single parent or living with partner

SES_it_: Socioeconomic covariates (e.g., income, education, employment)

u_i_: Random intercept for individual i (u_i_ ~ N(0, σ²))

## Annex A2: Detail description of Model Covariates

**Injury Severity**:
Injury severity was based on parent-reported injury status recorded in each wave of data collection. Injuries were categorised into three levels: no injury, injury without hospitalisation, and injury with hospitalisation. This classification was applied consistently across all waves for harmonisation.

**Cohort Membership**:
The study sample included two nationally representative cohorts from the LSAC: the B cohort, comprising children aged 0–1 years at baseline, and the K cohort, comprising children aged 4–5 years at baseline. Cohort membership was treated as a binary covariate to capture potential differences in cost profiles between the two groups.

**Lagged Injury Count**:
To account for the potential long-term cost impacts of prior injuries, a lagged injury variable was created based on each child’s history of injury reporting in earlier waves. Given the skewed distribution of these counts, the variable was categorised into four groups: no prior injury, 1–2 prior injuries, 3–5 prior injuries, and six or more prior injuries. This categorical approach was adopted to reduce the influence of outliers and to reflect the gradient effect of prior injury burden on healthcare costs.

**Asthma**:
The presence of asthma was captured through caregiver reports of whether the child had used prescribed asthma medication in the past 12 months. For early waves where this variable was not available, a related question on respiratory symptoms was used as a proxy. The data were harmonised across waves to create a consistent asthma covariate.

**Obesity**:
Children’s weight status was determined based on age- and sex-adjusted BMI classifications. Across waves, BMI categories were standardised into four groups: underweight, normal weight, overweight, and obese. For early waves where BMI data were reported as percentiles, appropriate cut-offs were applied to align with these categories.

**Disability**:
Disability status was represented through five binary indicators reflecting different types of reported conditions: sensory, physical, psychological, long-term health condition, and multiple disabilities. Where disability data were missing for a specific wave and cohort, a separate ‘missing’ category was created to retain those records in the analysis.

**Prior Injury History**:
A cumulative indicator of prior injuries was constructed by aggregating injury reports from earlier waves. The cumulative count was then categorised into 0, 1–2, 3–5, and 6+ injuries to capture overall exposure to prior injury events.

**Parental Proactiveness in Seeking Healthcare**:
This covariate was derived from two indicators: a baseline measure of difficulty affording medical care and a longitudinal measure of the caregiver’s perceived ability to raise emergency funds for healthcare. These variables were combined and categorised into three levels—high, moderate, and low proactiveness—to reflect financial readiness to seek medical care.

**Private Health Insurance**:
Private hospital insurance status was assessed at baseline. We assumed this characteristic to be stable during the study period and replicated its value across all waves for each individual. This allowed inclusion of the variable as a time-invariant covariate in panel regression models. Inclusion of this covariate helps to account for differences in healthcare access related to insurance coverage. This was included as a binary covariate.

**Remoteness**:
Remoteness of residence was classified according to the national geographic remoteness structure and categorised as urban, regional, or remote. This classification was maintained across waves to control for variation in service accessibility.

**Socioeconomic Status (SES)**:
SES was captured using a composite of household income quintile, maternal education level, and parental employment status. These indicators were harmonised across waves and included as categorical variables.

**Non-Injury Hospital Stay**:
Children’s hospitalisation for conditions unrelated to injury was included as a binary indicator. For the K cohort at wave 8, where this information was unavailable, missingness was coded as a separate category to retain those observations in the model.

Annex B: Mixed Effect GLM Regression Model Results

|  |  |  | | 95% CI | | | |  |
| --- | --- | --- | --- | --- | --- | --- | --- | --- |
| Model variables | Coefficient | P-value | | LB | | UP | |  |
|  |  |  | |  | |  | |  |
| Injury |  |  | |  | |  | |  |
| *No Injury* | Ref. |  | |  | |  | |  |
| *Injured without hospitalisation* | 0.204 | 0.000 | | 0.184 | | 0.224 | |  |
| *Injured with hospitalisation* | 0.637 | 0.000 | | 0.574 | | 0.700 | |  |
| Sex |  |  | |  | |  | |  |
| *Male* | Ref. |  | |  | |  | |  |
| *Female* | -0.010 | 0.445 | | -0.035 | | 0.015 | |  |
| Age | 0.014 | 0.000 | | 0.012 | | 0.016 | |  |
| Cohort |  |  | |  | |  | |  |
| *B cohort* | Ref. |  | |  | |  | |  |
| *K cohort* | -0.221 | 0.000 | | -0.247 | | -0.195 | |  |
| Family structure |  |  | |  | |  | |  |
| *Single parents* | Ref. |  | |  | |  | |  |
| *Both parents* | -0.065 | 0.000 | | -0.093 | | -0.038 | |  |
| Income Quintile |  |  | |  | |  | |  |
| *Quintile 1* | 0.055 | 0.000 | | 0.024 | | 0.086 | |  |
| *Quintile 2* | 0.046 | 0.003 | | 0.016 | | 0.076 | |  |
| *Quintile 3* | 0.032 | 0.027 | | 0.004 | | 0.061 | |  |
| *Quintile 4* | -0.004 | 0.748 | | -0.031 | | 0.023 | |  |
| *Quintile 5* | Ref. |  | |  | |  | |  |
| Mothers' education |  |  | |  | |  | |  |
| *Less than year 12* | Ref. |  | |  | |  | |  |
| *Year 12* | 0.023 | 0.300 | | -0.020 | | 0.066 | |  |
| *Professional qualification/certificate course* | 0.047 | 0.009 | | 0.012 | | 0.082 | |  |
| *Graduate/Diploma* | 0.070 | 0.000 | | 0.033 | | 0.106 | |  |
| *Post-graduate* | 0.060 | 0.023 | | 0.008 | | 0.111 | |  |
| Mothers' employment |  |  | |  | |  | |  |
| *Employed* | Ref. |  | |  | |  | |  |
| *Unemployed* | 0.060 | 0.021 | | 0.009 | | 0.110 | |  |
| *Not in labour force* | 0.050 | 0.000 | | 0.027 | | 0.073 | |  |
| *Not disclosed* | 0.076 | 0.258 | | -0.055 | | 0.207 | |  |
| Remoteness |  |  | |  | |  | |  |
| *Major cities* | Ref. |  | |  | |  | |  |
| *Inner region* | -0.128 | 0.000 | | -0.156 | | -0.101 | |  |
| *Outer region* | -0.211 | 0.000 | | -0.246 | | -0.175 | |  |
| *Remote/Others* | -0.309 | 0.000 | | -0.379 | | -0.239 | |  |
| *No of injury incident lagging in previous wave* | | |  | |  | |  | |
| No injury | Ref. |  | |  | |  | |  |
| 1-2 injuries | -0.042 | 0.000 | | -0.064 | | -0.019 | |  |
| 3-5 injuries | 0.067 | 0.051 | | 0.000 | | 0.134 | |  |
| 6+ injuries | 0.185 | 0.004 | | 0.058 | | 0.311 | |  |
| Non-injury related hospital stays | | |  | |  | |  | |
| No hospital stays | Ref. |  | |  | |  | |  |
| Had non-injury hospital stay | 0.702 | 0.000 | | 0.667 | | 0.736 | |  |
| Missing (K cohort Wave 8) | 0.400 | 0.000 | | 0.358 | | 0.443 | |  |
| Asthma/wheezing medication |  |  | |  | |  | |  |
| No | Ref. |  | |  | |  | |  |
| Yes | 0.265 | 0.000 | | 0.239 | | 0.290 | |  |
| Obesity Status |  |  | |  | |  | |  |
| Normal weight | Ref. |  | |  | |  | |  |
| Underweight | 0.035 | 0.067 | | -0.002 | | 0.072 | |  |
| Overweight | 0.068 | 0.000 | | 0.045 | | 0.091 | |  |
| Obese | 0.272 | 0.000 | | 0.240 | | 0.303 | |  |
| Missing | 0.187 | 0.000 | | 0.138 | | 0.236 | |  |
| Disabilities |  |  | |  | |  | |  |
| No disability | Ref. |  | |  | |  | |  |
| Sensory | 0.195 | 0.000 | | 0.141 | | 0.250 | |  |
| Physical | 0.241 | 0.000 | | 0.159 | | 0.322 | |  |
| Psychological | 0.690 | 0.000 | | 0.584 | | 0.796 | |  |
| Other long-term | 0.277 | 0.000 | | 0.232 | | 0.322 | |  |
| Multiple disability | 0.485 | 0.000 | | 0.432 | | 0.538 | |  |
| Private hospital insurance at baseline year | | |  | |  | |  | |
| No | Ref. |  | |  | |  | |  |
| Yes | 0.110 | 0.000 | | 0.084 | | 0.137 | |  |
| Parental proactiveness |  |  | |  | |  | |  |
| High proactiveness | Ref. |  | |  | |  | |  |
| Moderate proactiveness | -0.013 | 0.200 | | -0.033 | | 0.007 | |  |
| Low proactiveness | 0.042 | 0.009 | | 0.010 | | 0.073 | |  |
| No response | -0.067 | 0.285 | | -0.189 | | 0.055 | |  |

Annex C: Marginal effect (dy/dx) and CI across the different injury categories by age of children, considering non-injury as reference outcome*

| Age | B Cohort | |  | K Cohort | |
| --- | --- | --- | --- | --- | --- |
|  | Injuries WOH | Injuries WH |  | Injuries WOH | Injuries WH |
|  | dy/dx (95% CI) | dy/dx (95% CI) |  | dy/dx (95% CI) | dy/dx (95% CI) |
|  |  |  |  |  |  |
| 0 | 78 (70-87) | 308 (266-350) |  | 63 (56-70) | 247 (213-281) |
| 1 | 79 (71-88) | 312 (270-355) |  | 64 (57-71) | 251 (217-284) |
| 2 | 81 (72-89) | 317 (274-360) |  | 65 (57-72) | 254 (220-288) |
| 3 | 82 (73-91) | 321 (278-365) |  | 66 (58-73) | 258 (223-293) |
| 4 | 83 (74-92) | 326 (282-370) |  | 66 (59-74) | 261 (226-297) |
| 5 | 84 (75-93) | 331 (286-375) |  | 67 (60-75) | 265 (229-301) |
| 6 | 85 (76-95) | 335 (290-380) |  | 68 (61-76) | 269 (233-305) |
| 7 | 87 (77-96) | 340 (295-386) |  | 69 (62-77) | 273 (236-309) |
| 8 | 88 (78-97) | 345 (299-391) |  | 70 (63-78) | 277 (240-314) |
| 9 | 89 (79-99) | 350 (303-397) |  | 71 (64-79) | 281 (243-318) |
| 10 | 90 (81-100) | 355 (307-403) |  | 72 (65-80) | 285 (246-323) |
| 11 | 92 (82-101) | 360 (312-408) |  | 73 (66-81) | 289 (250-327) |
| 12 | 93 (83-103) | 365 (316-414) |  | 74 (66-82) | 293 (254-332) |
| 13 | 94 (84-104) | 370 (321-420) |  | 76 (67-84) | 297 (257-337) |
| 14 | 96 (85-106) | 376 (325-426) |  | 77 (68-85) | 301 (261-341) |
| 15 | 97 (86-107) | 381 (330-432) |  | 78 (69-86) | 305 (265-346) |
| 16 | 98 (88-109) | 386 (334-438) |  | 79 (70-87) | 310 (268-351) |
| 17 | 100 (89-110) | 392 (339-445) |  | 80 (71-88) | 314 (272-356) |
| 18 | 101 (90-112) | 398 (344-451) |  | 81 (72-90) | 319 (276-361) |
| 19 | 103 (91-114) | 403 (349-458) |  | 82 (73-91) | 323 (280-367) |

*Note: 1) dy/dx for factor levels is the discrete change from the base level, 2) Considering other covariates at their mean values.

Annex D: Marginal effects of healthcare costs (95% CI) across the covariates categories (sub-group analysis)

| **Covariates** | **Marginal Effects (dy/dx)** | **p-value** | **95% Confidence Interval** | |
| --- | --- | --- | --- | --- |
|  |  |  | Lower | Upper |
| Injury |  |  |  |  |
| *No Injury* | ref. |  |  |  |
| *Injured without hospitalisation* | 83.68 | 0.000 | 74.73 | 92.64 |
| *Injured with hospitalisation* | 329.04 | 0.000 | 285.16 | 372.93 |
| Sex |  |  |  |  |
| *Male* | ref. |  |  |  |
| *Female* | -3.81 | 0.445 | -13.59 | 5.97 |
| Age | 5.54 | 0.000 | 4.80 | 6.28 |
| Family structure |  |  |  |  |
| *Single parents* | ref. |  |  |  |
| *Both parents* | -26.05 | 0.000 | -37.30 | -14.80 |
| Income Quintile |  |  |  |  |
| *Quintile 1* | 21.58 | 0.000 | 9.49 | 33.67 |
| *Quintile 2* | 17.85 | 0.003 | 6.26 | 29.44 |
| *Quintile 3* | 12.48 | 0.027 | 1.39 | 23.56 |
| *Quintile 4* | -1.67 | 0.748 | -11.85 | 8.52 |
| *Quintile 5* | ref. |  |  |  |
|  |  |  |  |  |
| Mothers' education |  |  |  |  |
| *Less than year 12* | ref. |  |  |  |
| *Year 12* | 8.59 | 0.300 | -7.65 | 24.83 |
| *Professional qualification/certificate course* | 17.88 | 0.008 | 4.66 | 31.10 |
| *Graduate/Diploma* | 26.91 | 0.000 | 12.92 | 40.90 |
| *Post-graduate* | 22.99 | 0.024 | 3.07 | 42.92 |
| Mothers' employment |  |  |  |  |
| *Employed* | ref. |  |  |  |
| *Unemployed* | 23.66 | 0.025 | 2.97 | 44.36 |
| *Not in labour force* | 19.77 | 0.000 | 10.43 | 29.10 |
| *Not disclosed* | 30.21 | 0.276 | -24.13 | 84.55 |
| Remoteness |  |  |  |  |
| *Major cities* | ref. |  |  |  |
| *Inner region* | -49.70 | 0.000 | -60.16 | -39.25 |
| *Outer region* | -78.27 | 0.000 | -90.76 | -65.79 |
| *Remote/Others* | -109.63 | 0.000 | -131.25 | -88.00 |
| Cohort |  |  |  |  |
| *B cohort* | ref. |  |  |  |
| *K cohort* | -85.93 | 0.000 | -96.27 | -75.59 |
| *No. of injury incident lagging in previous wave* | |  |  |  |
| No injury | ref. |  |  |  |
| 1-2 injuries | -16.05 | 0.000 | -24.53 | -7.57 |
| 3-5 injuries | 27.21 | 0.058 | -0.98 | 55.39 |
| 6+ injuries | 79.56 | 0.009 | 19.91 | 139.21 |
| Non-injury related hospital stays |  |  |  |  |
| No hospital stays | ref. |  |  |  |
| Had non-injury hospital stay | 367.89 | 0.000 | 343.22 | 392.56 |
| Asthma medication/wheezing |  |  |  |  |
| No | ref. |  |  |  |
| Yes | 112.55 | 0.000 | 100.62 | 124.48 |
| Obesity Status |  |  |  |  |
| Normal weight | ref. |  |  |  |
| Underweight | 13.19 | 0.071 | -1.12 | 27.50 |
| Overweight | 26.20 | 0.000 | 17.08 | 35.31 |
| Obese | 116.43 | 0.000 | 101.46 | 131.40 |
| Missing | 76.83 | 0.000 | 54.99 | 98.67 |
| Disabilities |  |  |  |  |
| No disability | ref. |  |  |  |
| Sensory | 78.01 | 0.000 | 53.91 | 102.11 |
| Physical | 98.40 | 0.000 | 60.68 | 136.13 |
| Psychological | 380.56 | 0.000 | 301.71 | 459.42 |
| Other long-term | 117.05 | 0.000 | 95.55 | 138.55 |
| Multiple disability | 231.63 | 0.000 | 200.31 | 262.95 |
|  |  |  |  |  |
| Private hospital insurance at baseline year | |  |  |  |
| No |  |  |  |  |
| Yes | 43.14 | 0.000 | 32.70 | 53.57 |
| Parental proactiveness |  |  |  |  |
| High proactiveness |  |  |  |  |
| Moderate proactiveness | -5.05 | 0.199 | -12.76 | 2.66 |
| Low proactiveness | 16.66 | 0.010 | 3.94 | 29.38 |
| No response | -25.19 | 0.269 | -69.87 | 19.50 |

Annex E: Sensitivity Analyses

Table E-1: Sensitivity Analysis - Model Comparison Table

| Model | Coefficient: Injury w/o Hospital (SE) | Coefficient: Injury w/ Hospital (SE) | Random Effects Included | Model fit | | | | |
| --- | --- | --- | --- | --- | --- | --- | --- | --- |
|  |  |  |  | N | ll(model) | df | AIC | BIC |
| Mixed-effects GLM | 0.204(0.0103)^***^ | 0.639(0.0321)^***^ | Yes | 51,448 | -352551 | 43 | 705187.8 | 705568.2 |
| GEE (Gamma, log link) | 0.164(0.0138)^***^ | 0.603(0.0549)^***^ | No | 51,448 | NA | 41 | NA | NA |
| Fixed-effects (log-cost) | 0.227(0.0114)^***^ | 0.598(0.0398)^***^ | No (within-child) | 51,448 | -60822.6 | 37 | 121719.2 | 122046.6 |
| Pooled GLM | 0.182(0.0161)^***^ | 0.627(0.0161)^***^ | No | 51,448 | -357222 | 41 | 714525.9 | 714888.6 |

Notes: Standard errors in parentheses; *p<0.10, **p<0.05, ***p<0.01.

Table E-2: Sensitivity Analysis Results - Comparison of other covariates across the injury cost models

| Covariates | (1) Mixed-effects GLM (Gamma) | (2) GEE (Gamma) | (3) Fixed Effects (log cost) | (4) Mixed-effects GLM (Alternative Covariates) |
| --- | --- | --- | --- | --- |
|  | Coefficient (SE) | Coefficient (SE) | Coefficient (SE) | Coefficient (SE) |
| Non-injury hospital stays (Ref: No hospital stays) | 0.702*** (0.018) | 0.657*** (0.032) | 0.623*** (0.022) | 0.823*** (0.027) |
| Prior injuries (1–2) (Ref: No prior injuries) | -0.042*** (0.011) | -0.045*** (0.017) | -0.046*** (0.012) | -0.025 (0.018) |
| Prior injuries (3–5) (Ref: No prior injuries) | 0.067* (0.034) | 0.042 (0.053) | 0.034 (0.039) | 0.110** (0.053) |
| Prior injuries (6+) (Ref: No prior injuries) | 0.185*** (0.065) | 0.212 (0.152) | 0.094 (0.071) | 0.521*** (0.101) |
| Asthma/wheezing presence (Ref: No asthma/wheezing) | 0.265*** (0.013) | 0.226*** (0.021) | 0.227*** (0.018) | 0.284*** (0.017) |
| Overweight (Ref: Normal weight) | 0.068*** (0.012) | 0.072*** (0.020) | 0.080*** (0.015) | 0.059*** (0.017) |
| Obese (Ref: Normal weight) | 0.272*** (0.016) | 0.261*** (0.023) | 0.286*** (0.020) | 0.224*** (0.022) |
| Disability - Sensory (Ref: No disability) | 0.140** (0.063) | 0.116 (0.084) | 0.121 (0.072) | 0.121 (0.089) |
| Disability - Physical (Ref: No disability) | 0.329*** (0.070) | 0.281*** (0.088) | 0.276*** (0.074) | 0.333*** (0.096) |
| Disability - Psychological (Ref: No disability) | 0.690*** (0.054) | 0.613*** (0.077) | 0.580*** (0.066) | 0.762*** (0.083) |
| Disability - Other long-term (Ref: No disability) | 0.146*** (0.054) | 0.120 (0.078) | 0.121 (0.062) | 0.171** (0.079) |
| Disability - Multiple (Ref: No disability) | 0.483*** (0.061) | 0.442*** (0.085) | 0.456*** (0.069) | 0.532*** (0.088) |
| Parental proactiveness (moderate) (Ref: High proactiveness) | 0.118*** (0.013) | 0.091*** (0.019) | 0.092*** (0.015) | 0.138*** (0.017) |
| Parental proactiveness (low) (Ref: High proactiveness) | 0.255*** (0.018) | 0.201*** (0.025) | 0.213*** (0.019) | 0.288*** (0.024) |
| Cohort (K vs B) (Ref: B cohort) | -0.221*** (0.013) | -0.232*** (0.019) | – | -0.214***(0.014) |
| Constant | 5.530*** (0.026) | 5.558*** (0.045) | 5.128*** (0.040) | 5.535*** (0.031) |
| Observations | 51,448 | 51,448 | 51,448 | 51,448 |

Notes: Standard errors in parentheses; *p<0.10, **p<0.05, ***p<0.01.
